# Resolution of persistent SARS-CoV-2 infection with prolonged intravenous remdesivir and vaccination in a patient post CAR-T

**DOI:** 10.1007/s12185-022-03518-2

**Published:** 2023-02-09

**Authors:** Sai Ambati, Bazga Ali, Owen Seddon, Andrew Godkin, Martin Scurr, Catherine Moore, Clare Rowntree, Jonathan Underwood

**Affiliations:** 1 Department of Internal Medicine, Cardiff and Vale University Health Board, University Hospital of Wales, Cardiff, Wales, UK; 2 Department of Infectious Disease, Cardiff and Vale University Health Board, Cardiff, Wales, UK; 3 Department of Gastroenterology and Hepatology, Division of Infection and Immunity, School of Medicine, Cardiff and Vale University Health Board, Cardiff University, Cardiff, Wales, UK; 4 Division of Infection and Immunity, School of Medicine, Cardiff University, ImmunoServ Ltd, Cardiff, Wales, UK; 5 Department of Virology, Cardiff and Vale University, Cardiff, Wales, UK; 6 Department of Haematology, Cardiff and Vale University Health Board, Cardiff, Wales, UK; 7 Department of Infectious Disease, Division of Infection and Immunity, School of Medicine, Cardiff and Vale University Health Board, Cardiff University, Cardiff, Wales, UK

**Keywords:** SARS-CoV-2, Remdesivir, B cell ALL, Post BMT, CAR-T therapy

## Abstract

SARS-CoV-2 virus is a single-stranded enveloped RNA virus, which causes coronavirus disease. Most of the immunocompetent patients with SARS-CoV-2 infection do have mild to moderate respiratory illness; however, in immunocompromised patients, the course of infection is unpredictable with high rates of infectivity and mortality. So, it is important to identify the immunocompromised patients early and establish the course of treatment accordingly. Here, we describe a 25-year-old male with background of B cell ALL, post-BMT and CAR-T therapy who received treatment with remdesivir and vaccination and was followed up for six months from the onset of symptoms to post vaccination, which showed resolution of symptoms and improvement of immunological markers. Here, we review the literature concerning the course and treatment of SARS-CoV-2 infection aimed at achieving cure in this patient.

## Introduction

Management of SARS-COVID-19 is challenging as there is no single established and effective treatment protocol that accounts for immune status [1, 2]. There is an increased risk of mortality and morbidity from COVID-19 in immunodeficient adults compared to the general population [3, 4]. The pathology may differ between immunocompetent and immunodeficient patients, with the former hypothesized to have immune-mediated severe disease, whereas the latter may have more virally mediated pathology [5]. Most immunocompetent patients, even with severe disease respond to a short course of dexamethasone ± remdesivir and become PCR-negative, whereas immunocompromised patients may have prolonged PCR positivity and bouts of symptoms requiring intervention [6]. Patients post BMT with SARS-CoV-2 often have prolonged infections and poor treatment outcomes [7, 8]. Here, we report the prolonged use of remdesivir to treat persistent COVID-19 in a patient with B Cell ALL who had undergone previous allogenic stem cell transplantation and CAR-T therapy.

## Case Report

A 25-year-old male patient presented to the Hematology Department with a two-week history of fever, dry cough and breathlessness on minimal exertion. His oxygen saturation on room air was 94%. His background includes Ph-negative B-ALL diagnosed in 2011 and was treated with UKALL regime B (VCR, DEX, PEG ASP, DNR/DOX, CYCLO, ARA-C, and MTX). He had a relapse in July 2018 with cerebral and renal masses, marrow infiltration, for which he received FLAG-Ida, NOPHO Block B, brain RT consolidated with MDU Cyclophosphamide with TBI(Cy/TBI) and allogenic stem cell transplant in December 2018. He had a second relapse in July 2020 with enlarging renal lesions on CT and bone marrow disease for which he had CAR-T therapy (Chimeric Antigen Receptor T Cell therapy) six months before his presentation. He had evidence of B and T cell dysfunction and was receiving *Pneumocystis jirovecii* (PJP) and HSV prophylaxis and had monthly immunoglobin replacement.

**Fig. 1:**
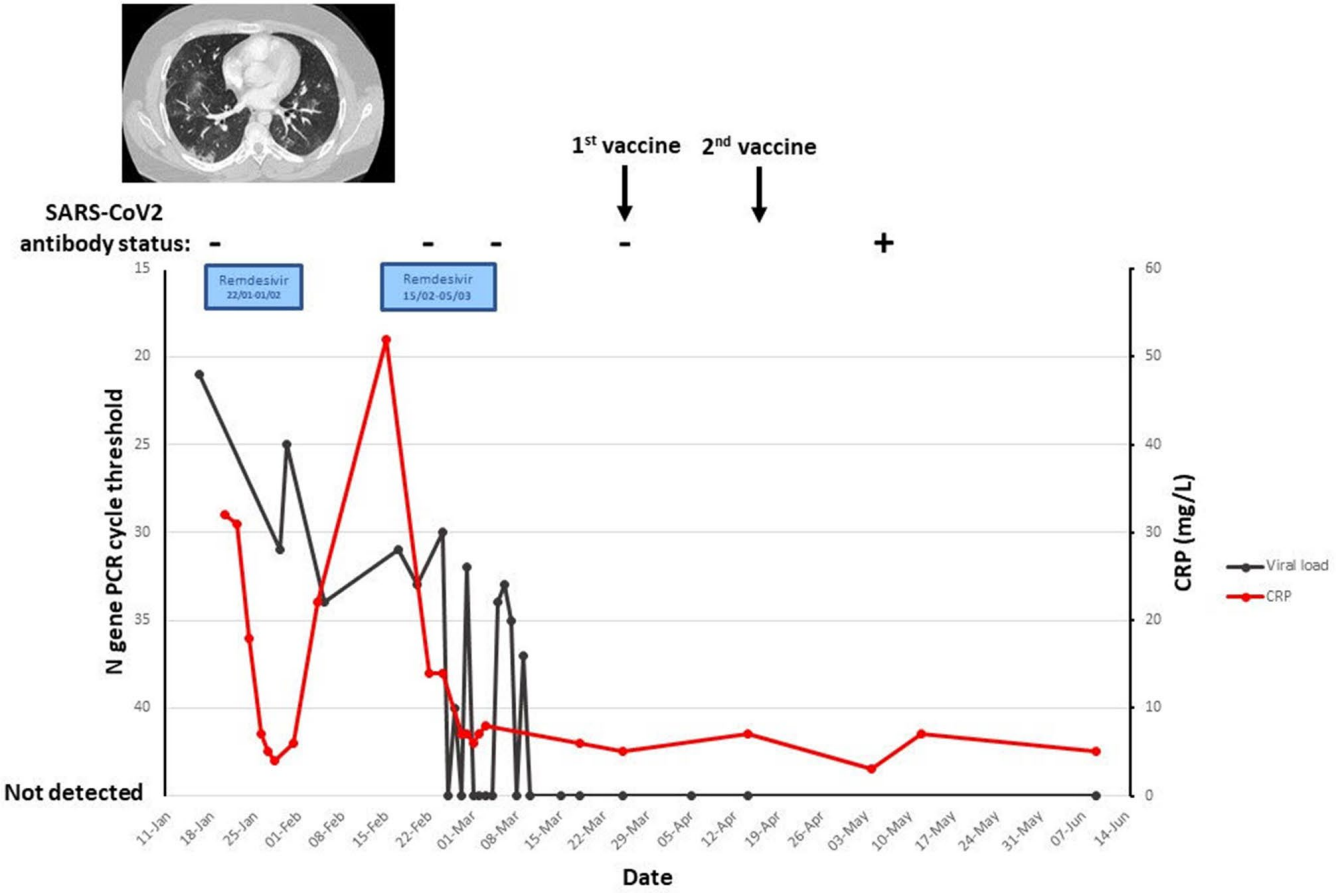
This graph depicts the improvement of inflammatory markers (indicated in red) and no detection of SARS-CoV-2 on PCR/viral load (indicated in black). After 10-day course of remdesivir, there is significant improvement in the CRP. The symptoms reappeared with worsening of inflammatory markers, which improved with second course of remdesivir. With subsequent administration of vaccine, there was no detection of the SARS-CoV-2 on the PCR

Investigation at this admission revealed him to be positive for SARS-CoV-2 by a PCR test on pharyngeal/nasal secretions. CT pulmonary angiogram (CTPA) showed bilateral, patchy sub-pleural linear and ground glass opacification in keeping with COVID-19 pneumonitis (figure one). Pan respiratory PCRs (Luminex) did not show any evidence of viral co-infection and PJP PCR and β-D-glucan were negative. He symptomatically improved and became afebrile after 24 h of remdesivir. His symptoms fully resolved, although he remained PCR-positive on dry throat swab, albeit with some evidence of viral clearance with his SARS-CoV-2 PCR cycle threshold (Ct) increasing. He completed a 10-day course due to his immunodeficiency and was discharged (Fig. 1).

The patient re-presented to the Emergency Unit 3 days after discharge with recurrence of fever and cough, but without significant hypoxia, and he did not appear breathless. The fever resolved with paracetamol and he was discharged home. He was readmitted 10 days later due to persistent and worsening fevers and cough. Repeat CT demonstrated new areas of pneumonitis. An infection screen did not reveal any concurrent infections. He was SARS-CoV-2 antibody-negative [Euroimmun, London]. However, a whole blood SARS-CoV-2 T cell assay (measuring IFNγ) [ImmunoServe Ltd., Cardiff] was deemed positive (IFNγ: 106 pg/ml, negative control < 23 pg/ml.) [9–11]. Remdesivir was re-administered, which again resulted in rapid symptomatic improvement. He received an additional 18 days of remdesivir pending a decision on the compassionate use of convalescent plasma or casirivimab/imdevimab (both of which were ultimately declined). This treatment course resulted in symptom resolution and normalization of inflammatory markers and radiology (Fig. 1). SARS-CoV-2 PCR cycle thresholds again increased with treatment and became negative (Fig. 1). Remdesivir was well tolerated other than an asymptomatic rise in his ALT which normalized after cessation of therapy.

**Fig. 2:**
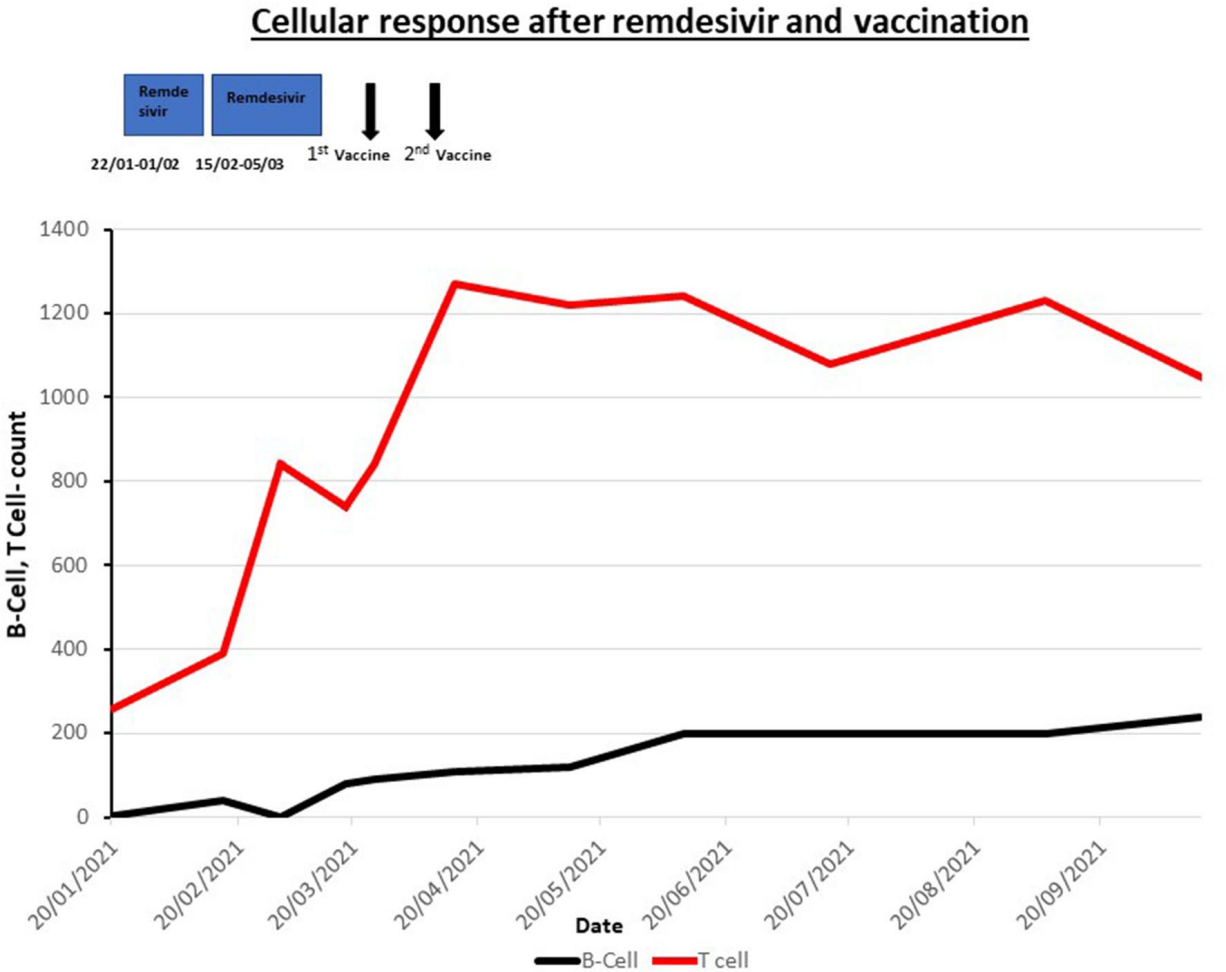
The graph indicates the cellular response with gradual improvement in the B cell (represented in black) and T cell (in red) counts after treatment with remdesivir and two doses of vaccination

Despite prolonged PCR positivity, SARS-CoV-2 antibodies were repeatedly not detectable in the patients’ serum. Despite the lack of antibodies, the presence of SARS-CoV-2-specific T cells, and the suppression of viral replication with remdesivir, encouraged us to administer a SARS-CoV-2 vaccine with Pfizer/BioNTech mRNA vaccine and tolerated it well without developing cytopenias or cytokine release syndrome. A repeat T cell assay was strongly positive for anti-SARS-CoV responses (> 1000 pg/ml) at the time of first vaccination and again three weeks after completing the primary course. The T cell and B cell counts gradually improved after remdesivir treatment and vaccination, suggesting a good cellular response (Fig. 2). Having been repeatedly SARS-CoV-2 antibody-negative (figure one), he seroconverted three weeks after completing his primary vaccination course. He remained well and SARS-CoV-2 PCR-negative since.

## Discussion

Here, we describe a prolonged treatment course of remdesivir associated with sustained PCR negativity (i.e., cure) in a patient with significant secondary immunodeficiency, following CAR-T therapy. Remdesivir is a nucleoside analog which targets RNA-dependent RNA polymerase (RdRp) and inhibits the replication of SARS-CoV-2 [12]. The evidence of benefit in patients with COVID is limited and there is some controversy over its utility [13]. However, these studies almost exclusively recruited immunocompetent participants where the disease process may differ compared to the patients with immunodeficiency. Here, remdesivir resulted in rapid symptomatic improvement of SARS-CoV-2 infection with an associated increase in Ct implying improvement was mediated by a reduction in viral load. However, despite evidence of a T cell response to SARS-CoV-2 comparable to healthy controls, he did not seroconvert and had a clinical relapse within days of stopping a 10-day course of remdesivir associated with an increased viral load. The rapid recrudescence in this case highlights the importance of inhibiting viral replication to achieve clearance and suggests that treatment courses longer than 10 days or treating until sustained PCR negativity may be necessary in patients with significant immunodeficiency. This is supported by other case reports [13].

Following clinical cure and virological clearance, he remained seronegative despite robust T cell SARS-CoV-2 responses. He subsequently seroconverted following vaccination with Pfizer BioNTech mRNA vaccine, demonstrating the importance of vaccination, despite natural infection, in patients with immunodeficiency [14].

Antibody therapy with either convalescent plasma of monoclonal antibodies was not available in this case, and cure was attained with a prolonged course of remdesivir alone. However, given the potency of monoclonals, such as casirivimab/imdevimab, and beneficial results from clinical trials in hospitalized and non-hospitalized patients with SARS-CoV-2 infection [15], it is likely combination therapy with remdesivir in patients with significant immunodeficiency would be optimal in terms of chance of cure and prevention of the emergence of resistance given their different therapeutic targets.
